# Design and Rationale of a Two-Armed Randomized Controlled Trial on Yoga/Brisk Walking-Based Lifestyle Modification on Dementia Risk Reduction, and Influence of ApoE Genotypes on the Intervention

**DOI:** 10.14283/jarlife.2024.5

**Published:** 2024-05-15

**Authors:** M. Singh, V. Majumdar

**Affiliations:** 1. Swami Vivekananda Yoga Anusandhana Samsthana, Bangalore, Karnataka, India-560105.

**Keywords:** Dementia, lifestyle modification, randomized control trial, ApoE, middle-age

## Abstract

**Background/Introduction:**

Though considered a late-onset disease, the 2020 report of the Lancet Commission emphasizes the necessity of conducting primary prevention trials with an approach of never too early in the life course for dementia prevention. Driven by the same notion, we hereby aim to compare the dementia risk reduction potential of two potential interventions, 48 weeks (12 months) of yoga and brisk walking, in middle-aged high-risk subjects.

**Design:**

A randomized controlled trial.

**Setting:**

Community in India.

**Participants:**

In total, 323 at-risk dementia subjects will be recruited from community settings through health awareness camps and door-to-door surveys across Delhi, India. Participants will be randomized into yoga or brisk-walking groups (1:1). The yoga intervention group will receive 60 contact yoga sessions per 60-min/day at the community parks, followed by continued tele-supervised home practice, further followed by at-home self-practice, and will be tested at 3-time points (baseline, 24-week and 48-week, post-randomization) to test the efficacy of the intervention. The control group will be asked to do brisk walking daily for 45 minutes at their convenience, followed by weekly telephone follow-ups. Applying the intention-to-treat principle, the primary endpoint will be the change from baseline at the 12th month in the Cardiovascular Risk Factors, Aging, and Dementia (CAIDE) Scores. Secondary outcomes will include the composite scores derived from a comprehensive neuropsychology battery, comprising the Trail Making Test, Digit Span Test, N Back, Color Trail, Animal Fluency Test, COWA (Controlled Oral Word Association Test), and Digit Symbol Substitution. The primary outcome will be analyzed using mixed-effect models for repeated measures, adjusted for covariates as fixed effects. The study has been prospectively registered (CTRI/2023/02/049746) on February 15, 2023. The protocol was conceptualized in 2021 and approved by the Institutional Ethics Committee of SVYASA. Recruitment began in February 2023 and is underway with patient enrollment.

**Conclusion:**

To our knowledge, this is the first controlled trial to investigate the longitudinal effects of a yoga-based intervention on dementia risk reduction using the CAIDE risk score. The findings of this trial will also provide insight into a better understanding of genotype-dependent responses to yoga intervention and open up avenues for understanding the implications of gene-intervention interactions for precision prevention using yoga.

## Introduction

**T**he global trends of population aging have tremendously impacted life expectancy in the Southern Asian region (1). India, the most populous country in the world, is at an alarming stage of population aging, with an estimated share of 20% of individuals aged 60 years or older by 2050 (2). Increased dementia is one of the primary consequences of population ageing. The latest estimates by the Longitudinal Aging Study in India (LASI) indicate a 7.4% prevalence of dementia, with 8.8 million individuals being afflicted (3). Dementia refers to a diverse range of conditions, with Alzheimer’s disease and vascular dementia being the most prevalent types (4). Unfortunately, there are no effective therapies available yet to treat dementia. Positively, the pathological model of dementia provides an optimal window for its prevention given the lengthy course of its duration, which takes several years to emerge (5). Hence, early and accurate identification of people at high risk of dementia is critical for the effective implementation of preventive measures. The importance of modifiable risk factors highlights the risk of dementia (5). Detecting changes in these risk factors before the disease manifests clinically allows for timely and careful management of vascular risk factors, thereby delaying the onset of the disease (5). The Lancet Commission on Dementia Prevention, Intervention, and Care Report states that up to one-third of dementia cases are preventable, considering the key potentially reversible risk factors.

Non-pharmacological physical activity-based therapies have shown potential for reducing the risk of dementia (6, 7). The prevention model for dementia is based on the estimation of risk reduction using the Cardiovascular Risk Factors, Ageing, and Dementia (CAIDE) score. This score was developed to address the increasing need for early detection in the treatment of neurodegenerative diseases. The CAIDE score is a validated tool for estimating dementia risk derived from age, sex, education, systolic blood pressure, body mass index, serum total cholesterol, and physical activity in middle-aged community subjects and has been validated to estimate the risk of dementia 20 years later (10). However, the reported associations between physical activity and dementia risk reduction have not been established using the intervention. Mechanistically, exercise and physical activity-based interventions have been proposed to possibly work via attenuating the vascular risk and associated vascular cognitive impairments, thereby halting the advancement of neurodegenerative diseases and dementias. Yoga has a mechanistic basis that can effectively manage cardiovascular risk factors (8). We deemed that testing the same over a composite score like the Cardiovascular Risk Factors, Ageing, and Dementia (CAIDE) score would aid in the translation of the existing evidence to support the preventive potential of yoga for dementia. We also hypothesize a concomitant improvement in cognitive functions.

The causes of dementia are varied, but genetic variations in the apolipoprotein E (ApoE) gene have been identified as major factors contributing to the disease. This conclusion comes from extensive genome-wide association studies (GWASs) (11), which have been confirmed by studies in different populations worldwide, including Asian Indians (12, 13). ApoE plays a key role in both cerebral and peripheral cholesterol metabolism and underlies many functions involved in brain amyloid metabolism, blood-brain barrier integrity, and transport of various lipid species to neuronal cells, as well as in hepatic uptake of triglyceride-rich lipoproteins (13). Interestingly, the genotype of ApoE has also been reported to play an important role in response to interventions (14). ApoE is a significant genetic component that has been validated across many cohorts around the globe, including the Indian population (15). Exercise has been reported to be more beneficial in ApoE4 carriers than non-carriers towards amyloid deposition (16). To our knowledge, no study has explored the risk reduction associated with adherence to yoga and its plausible modification by ApoE ε4 allele status.

### Study Design

**Figure 1:**
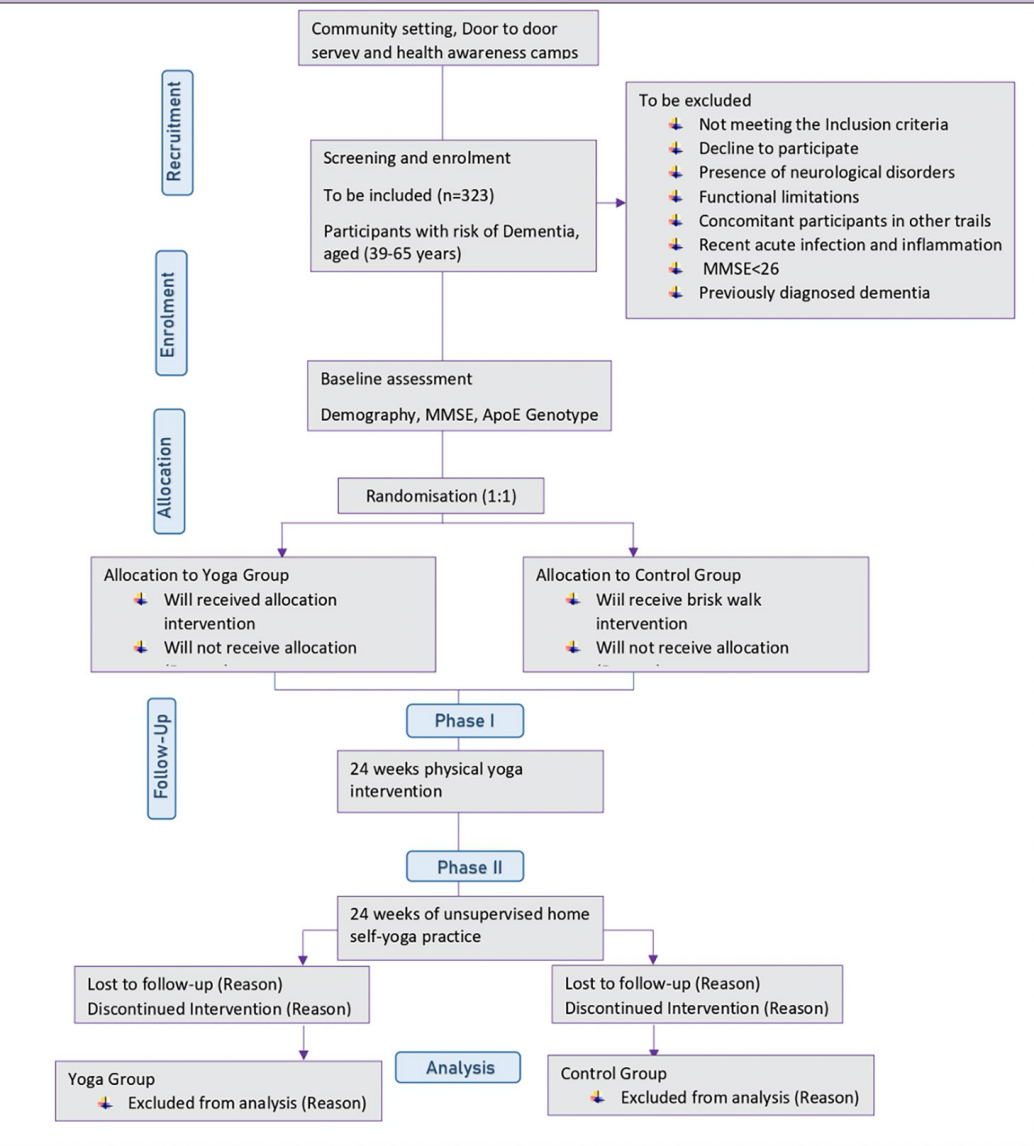
Participant flow chart for parallel design, based on the Consolidated Standards of Reporting Trials guidelines for transparent reporting of trials. CAIDE: cardiovascular risk factor, aging, and incidence of dementia

The study DERRY (Dementia Risk Reduction using yoga will be a prospective, parallel-group, single-center, and 2-arm randomized controlled trial wherein the subjects will be randomly allocated to two groups: yoga or active walking control. The protocol has been drafted following the CONSORT guidelines (Consolidated Standards of Reporting Trials) (17) (Figure 1). The study will be conducted between February 2023 and October 2024. A total of 323 adults (aged 30-65 years) with dementia risk (CAIDE score≥6) will be recruited from the community set up through door-to-door surveys and dementia awareness camps across Delhi, India. Eligible participants will be randomized into 1:1 yoga or active control groups. Primary and secondary outcomes will be assessed at baseline (before intervention) and at 24 and 48-week visits (with a 60-day window of flexibility) (see Figure 1). All participants will follow their usual medical regime during the study period. This protocol (RES/IEC-SVYASA/243/2022) has been approved by the Ethics Committee of Swami Vivekananda Yoga Anusandhana Samsthana. Interested and eligible individuals will be asked to attend an orientation session, and informed consent will be obtained from them before participation.

MMSE: mini mental status examination

## Recruitment and screening

Participants will be enrolled using convenience sampling between February 2023 and October 2024. Middle-aged individuals, aged 39 to 65 years, will be eligible if they have not regularly practiced any form of yoga in the previous 6 months and do not have a history of dementia. Patients will undergo a Mini-Mental State Exam (MMSE) with a score of at least 26 to rule out gross dementia. Additionally, they will be assessed using a CAIDE Risk Score (Cardiovascular Risk Factors, Aging, and Dementia) with a score of at least 6 points (18). Further, they will be excluded based on i) the presence of any neurological disorder; ii) significant arthritis; iii) recent acute infection or other inflammation; iv) persistent cognitive impairment due to psychoactive substance use; and v) individuals who have functional limitations that prevent them from walking or doing yoga and who have been advised against exercising by their physician or have undergone recent surgical interventions. Upon enrollment in the study, participants will be followed for 48 weeks (12 months) or until they withdraw from the study.

### Study outcomes and assessments

#### Primary outcome

The primary objective of the study is to assess the efficacy of a yoga intervention vs. brisk walking at 48 weeks on the risk reduction of dementia. The primary endpoint is a difference in the CAIDE risk scores between the yoga and brisk walking groups after 48 weeks (12 months). As shown in CONSORT, assessments will be conducted at baseline, 24 and 48 weeks over 365 days with a 60-day flexibility window (Table 1). However, the primary time endpoint represents a longer-term outcome.

**Table 1:** Schedule of enrolment, interventions, and assessments, according to SPIRIT 2013 guidelines

Time points	Screening Baseline	Intervention	Post-intervention	Endpoint
	week 0+2	24 weeks	24 + 1 week	48 + 1-week
Eligibility screening	x			
Informed consent	x			
Allocation	x			
***Intervention***		x		
Yoga/walking		x		
*Yoga*				
Phase I: supervised sessions		x		
Phase II: a synchronized yoga home practice		x	x	
*Control*		x	x	
Brisk walk: Weekly monitoring of daily steps				
*Assessments*				
Demographics and ApoE Genotype	x	x	x	x
***Primary Outcome***				
CAIDE dementia risk score	x	x	x	x
***Secondary Outcomes***				
Individual scores of CAIDE components				
*Cognitive assessments*				
TMT-A and B scores				
Digit Span Test scores	x	x	x	x
Verbal N Back	x	x	x	x
Color Trail	x	x	x	x
Animal Fluency Test	x	x	x	x
COWA	x	x	x	x
DSST	x	x	x	x
*Sleep Quality*				
Eligibility screening	x			
Informed consent	x			
Allocation	x			
***Intervention***		x		
Yoga/walking		x		
*Yoga*				
Phase I: supervised sessions		x		
Phase II: a synchronized yoga home practice		x	x	
Pittsburgh Sleep Quality Index (PSQI)				
Adherence		x	x	x

CAIDE- Cardiovascular risk factor, aging, and incidence of dementia; TMT- Trail making Test; DSTT- Digit symbol substitution test, COWA- Controlled Oral Word Association Test, ApoE- Apolipoprotein E

The research staff will evaluate the CAIDE risk score during screening. The CAIDE risk score ranges from 0 to 18, with higher scores indicating an increased risk of developing dementia (18), with scores of 8–9 indicating a 4.2% risk of developing dementia in the next 20 years (18, 20, 21). CAIDE is a comprehensive tool for predicting dementia risk in middle-aged individuals. The CAIDE Risk Score incorporates various non-modifiable and modifiable factors, including age, education, blood pressure, cholesterol levels, body mass index (BMI), physical activity, and ApoE status (18).

#### Secondary Outcomes

Secondary outcomes will include individual scores of CAIDE components and assessment scores of cognitive tests for memory, attention, language, verbal fluency, and executive ability, measured by neuropsychological tests or other objective measurements. The secondary clinical outcome of this study focuses on the 48-week change in the composite score of a comprehensive neuropsychology battery.

### Assessments

At the baseline and 48-week visits, the CAIDE score will be calculated using data on age, gender, self-reported years of formal education, systolic blood pressure, BMI, total cholesterol, physical activity, and ApoE status. Each component of the CAIDE score will undergo assessment, and a predetermined set of points will be allocated to each category of risk factors (17). The CAIDE score for each participant will be computed by summing up the points assigned to each risk factor category (18). Participants self-reported demographic details and physical activity levels, while healthcare professionals measured objective factors like systolic blood pressure, BMI, and blood cholesterol levels. The physical activity index was calculated based on activity duration and intensity. Genetic testing for ApoE ε4 carrier status was conducted during a specific exam. The CAIDE score, ranging from 0 to 18, will be used in a male-only cohort, with observed scores from 1 to 15 (18, 19).

At the outset, supplementary information encompassing demographic data and contemporary risk factors such as hypertension, diabetes, head injury, smoking, alcohol consumption, dyslipidemia, etc. will be documented for the participants.

This battery includes well-established tests to assess the global cognitive ability index as well as various domains of cognition such as memory, attention, language, verbal fluency, and executive ability, which will be measured by neuropsychological tests such as the Trail Making Test, Digit Span Test, N Back, Color Trail, Animal Fluency Test, COWA, and Digit Symbol Substitution.

In addition to the neuropsychology battery assessment, participants will also be asked to complete questionnaires to assess their quality of sleep using the Pittsburgh Sleep Quality Index (PSQI) and their overall quality of life using the World Health Organization Quality of Life (WHOQOL-BREF) questionnaire. These validated questionnaires provide valuable insights into participants’ subjective experiences of sleep quality and their overall well-being across physical, psychological, social, and environmental domains.

Furthermore, genotyping will be performed using DNA amplification through real-time PCR. This genetic analysis aims to explore potential associations between specific genetic variations and cognitive outcomes, providing insights into the role of genetics in cognitive function and dementia risk (22).

### Executive Function

#### Trail-making test

Trail-making test: A neuropsychological test of visual attention and task switching is called the Trail Making Test (23). There are two parts to it; part A seems to primarily depend on the effectiveness of visual scanning and psychomotor speed. In which the subject is asked to accurately connect a sequence of 25 dots as quickly as they can. Part B has circles with both numbers (1–13) and letters (A–L); the subject is instructed to connect the circles in an ascending pattern by alternating between the numbers and letters (i.e., 1-A-2-B-3-C, etc.) (23). Specifically, mental flexibility and a higher demand for working memory are required for executive control in TMT B. It is also capable of accurately identifying many cognitive disorders, including dementia and Alzheimer’s disease (23).

### Working Memory: Digit Span test, Verbal N Back

Digit Span: The original digit span task was similar to the Wechsler Memory Scale (24). The participant will be instructed to be attentive to a series of random numbers that will be played once per second. The subject was asked to recall the numbers in reverse order for the digit span backwards (DSB), while the subject was asked to repeat the numbers in a forward series for the digit span forwards (DSF) (25). Each correctly repeated series began with a two-number series and ended with a one-digit series. If the subject failed the first time, they were given another chance with a new set of random numbers for each sequence. If the test subject fails again after the second try, the test will be stopped, as will the longest series they attempt (25).

Verbal N back: Several Indian languages share thirty consonants, which are pronounced one per second. Thirty consonants total, nine of which are repeated (25). The repeated consonants are selected at random. In the 1-back test, the subject responds by tapping the table whenever a consonant is repeated consecutively. In the 2-back test, the subject responds by tapping the table whenever a consonant is repeated after an intervening consonant (26). Scores were based on the number of successes and failures on each test. A negative score was assigned based on the number of errors. After this, the overall score was determined (26).

### Attention: Color Trail Test

The Color Trails Test (CTT): The Color Trail Test is a language-free version of the Trail Making Test (TMT) that was developed to allow for broader cross-cultural application to measure sustained attention in adults (27). Numbered circles are printed with vivid pink or yellow backgrounds that are perceptible to color-blind individuals. For Part 1, the respondent uses a pencil to connect circles rapidly numbered 1–25 in sequence. For Part 2, the respondent rapidly connects numbered circles in sequence but alternates between pink and yellow (28).

### Language: Animal fluency test, COWA

Animal fluency test: The subject will be asked to come up with as many animal names as they can in a minute. The subject is instructed to omit any mention of fish, snakes, or birds. The score was formed by the number of names produced (26).

COWA: Frontal lobe dysfunction can hinder a person’s ability to quickly form words. A typical neuropsychological test for verbal fluency is the Controlled Oral Word Association Test (COWAT), also called the “FAS.” Three-word criteria make up the COWAT. The subject’s objective is to come up with as many words as possible that begin with the specified letter (F, A, or S) in one minute (29). Additionally, subjects are told to avoid using proper nouns, numbers, and the same word with a different suffix (29). Frontal lobe impairment has been successfully detected using the COWAT and other verbal fluency tests. Jerry Janowsky, Arthur Shimamura, and Larry Squire discovered in 1989 that individuals with circumscribed left or bilateral frontal lobe lesions produced noticeably fewer words than control subjects (30).

### Processing Speed: Digit Symbol Substitution

Digit Symbol Substitution: We will administer the DSST to capture processing speed. In this task, participants see a table with a mapping between nine symbols and the digits 1–9. The participants are given 9 seconds to fill in the respective numbers that correspond to the symbols in a large list of symbols. The dependent variable is how many symbols are successfully associated with the respective number (31, 32).

### Quality of Sleep

Pittsburgh Sleep Quality Index (PSQI); Improvement in the quality of sleep using PSQI Score after 6 weeks of intervention (33). The PSQI is a self-reported instrument that measures the quality of sleep as well as sleep disturbances over one month. The scale assesses seven domains: sleep quality, sleep duration, sleep latency, habitual sleep efficiency, sleep disturbances, use of sleep medication, and daytime dysfunction (33).

### Quality of life

World Health Organization Quality of Life (WHOQOL-BREF); improvement in the quality of sleep using the WHOQOL-BREF Score after 6 weeks of intervention. WHOQOL-BREF is a comprehensive tool for assessing quality of life. It was standardized with 26 items and developed by WHO (34). The scale provides a measure of an individual’s perception of quality of life for the four domains: (1) physical health (seven items), (2) psychological health (six items), (3) social relationships (three items), and (4) environmental health (eight items). In addition, it also includes two questions for the ‘overall quality of life’ and ‘general health’ facets. The domain scores are scaled in a positive direction (i.e., higher scores denote a higher quality of life). The range of scores is 4–20 for each domain (34).

### Mini-mental state evaluation score (MMSE)

The MMSE is a measure used for cognitive screening in both clinical and research areas. Scores ≤ 26 on the MMSE indicate the absence of severe dementia (35, 36).

### Genotyping of ApoE ε4

Genomic DNA will be extracted from 2 ml of human whole blood using the NucleoSpin® Blood L kit (Macherey-Nagel) according to the manufacturer’s instructions. Following this, genotyping of ApoE variants will be performed. To perform genotyping, we will use the primers reported by Calero (22). The forward primers are from positions 2886 to 2903, whereas the reverse primers are from positions 3041 to 3058. The polymorphic site contains the 3-end nucleotides, and specific primers will be designed to match one of the two variants at the ApoE positions 2903 and 3041. The primers will be combined in three reaction mixtures that are anticipated to produce an amplification product of 173 bp. These three reaction mixtures are «Reaction ApoE 2» (primers ApoE 112C and ApoE 158C), «Reaction ApoE3» (primers ApoE 112C and ApoE 158R), and «Reaction ApoE4» (primers ApoE 112R and ApoE 158R). Each PCR reaction mixture contained the following: 1× Power SYBR® Green PCR Master Mix (Applied Biosystems), 0.3 M of each primer, and 50 ng of genomic DNA. Negative controls will be performed by using the same reaction mixtures without DNA. All the reactions will be run in duplicate. The PCR amplification protocol would be as reported: initial AmpliTaq Gold DNA Polymerase activation at 95 ◦C for 10 min, followed by 40 cycles with denaturation at 95 ◦C for 15 s, and annealing + extension at 62 ◦C for 1 min. Amplification will be performed either on a 7500 Real-Time PCR System (96-well format) (Applied Biosystems) using the comparative Ct (Ct) method (22).

## Randomization and blinding

A computer-based program will be utilized by a statistician to generate a randomized list. Participants will be randomized by the system into blocks of four, six, eight, or ten, stratified by. Only research staff will be able to retrieve the generated list, which will be kept confidential. The participant will receive the allocation of intervention only after completion of their baseline assessment. Due to the nature of yoga or walking interventions, participants could not be blinded to the intervention allocation. However, outcome assessments will be carried out by blinded.

## Adherence

Adherence will be calculated based on participation in sessions. The trainer will maintain the logbook in Phase 1, while individual participants will receive notepads to keep records in Phase 2. All these will be collected during follow-up assessments. Attendance rates will be calculated by dividing the number of sessions attended by the total number of sessions. Overall attendance will then be calculated by averaging all participants’ attendance rates. Attrition rates will be calculated by dividing the number of individuals who dropped out of the study by the total number of enrolled participants. The reasons cited for study dropout will also be summarized.

### Intervention

Participants in the yoga group will be given intervention for 6 months, 5 days a week. Intervention would be delivered by qualified yoga professionals (Table 2). The participants will be trained to perform yoga practices by qualified yoga instructors. In the initial orientation at the community center, participants will be given 1 hour for each group-based sessions, which will consist of a 2-minute introduction followed by 10 minutes of gentle loosening movements followed by 1 minute of relaxation, 15 minutes of postures followed by 2 minutes of relaxation, followed by breathing practices of 15 minutes, and end with guided meditation (5 minutes) followed by 3 minutes of relaxation and a 2-minute closing prayer followed by 5 minutes of query and discussion. Overall, the adjunct yoga intervention will be divided into 2 phases: Phase 1, including supervised sessions at the community center, including the orientation; and Phase 2 unsynchronized teleyoga at-home practice (120 sessions) (Table 1). There would be 24 weeks of yoga training followed by asynchronous teleyoga home practice for 24 weekss, 5 days a week.

**Table 2:** Schedule of Yoga Sessions

Yoga Sessions		
	Supervised by Yoga Instructor	Home-Based Asynchronized Teleyoga Practice
Period	Only during Phase I	During Phaes II
Number of sessions	120	120
Duration/ session	60 min	60 min
	Welcome and Philosophical perspective (2 min) Preparatory loosening exercises (10 min) + IRT (1 min) Postural yoga Asanas (15 min) + relaxation (2 min) Breathing exercises: Pranayama (15 min) Guided meditation (5 min) relaxation (3 min) Closing prayer (2 min) Query and discussion (5 min)	Welcome and Philosophical perspective (2 min) Preparatory loosening exercises (10 min) + IRT (1 min) Postural yoga Asanas (15 min) + relaxation (2 min) Breathing exercises: Pranayama (15 min) Guided meditation (5 min) relaxation (3 min) Closing prayer (2 min) Query and discussion (5 min)

The standardized and validated yoga-based intervention module, including çithilikaraa vyäyäma (loosening practices), äsana (postures), pranäyama (breathing practices), and dhyäna (meditation), aimed at bringing harmony to mind and body, will be provided to the participants. While delivering the intervention, the therapist will observe the condition of each subject and make sure to make them understand each practice by adding more explanations to each approach. Modifications will be made individually, according to each participant’s specific limitations. We will be using the previously published module of yoga intervention already reported to reduce cardiovascular risk factors such as high blood pressure and dyslipidemia. The authors reported that using the specific module titled Integrated Yoga Therapy there would be significant improvements in baroreflex sensitivity, systolic blood pressure, and total peripheral vascular resistance in hypertensive patients (37). In a similar vein, Sharma et al. (2020) reported on the management of lipid profiles in patients with coronary artery disease (CAD) using IAYT (38).

We also aim to involve a few strategies to reduce attrition and minimize loss to follow-up based on our prior experiences with yoga-based interventions: (a) being responsive to participants and/or spouse/care partner questions; and (c) the yoga coordinators will meet with participants throughout the study, sometimes traveling to the participant’s fitness facility or home, which should enhance adherence to the program and allow the development of a strong researcher-participant relationship. We have included periodic tele-synchronized sessions as well to decrease the travel burden on participants considering vacations, work commitments, or other reasons influencing adherence to the trial. We have included participants to choose where they exercise and provide the means to do so to promote retention and long-term adherence to exercise. A research coordinator will contact the participants to inquire about their current health status at least once per month.

### Statistical analysis

Sample size: A sample size of 269 (n = 135; 135) was derived based on a moderate effect size assumption, using G power software’s formula for F-test, ANCOVA with fixed effect, main effect and main effect interaction model for 2 group comparisons. The calculated sample size also aligns with the estimate presented by Leon et al. 2009 (39). Further, assuming an attrition rate of 20%, the final sample size is n = 323 subjects randomized in a 1:1 ratio to yoga or brisk walking.Baseline characteristics will be presented using appropriate descriptive statistics. Before analysis, variable distributions will be examined to ensure that assumptions of normality are met using statistical software (SPSS, Statistical Package for the Social Sciences, Version 20.0) with the Shapiro-Wilk test. The equivalence of variance will be found using the F distribution test. Depending on the distribution of data, parametric or nonparametric tests will be performed within and between group comparisons for baseline data. The baseline characteristics of the study completers will also be compared with drop-outs. If the data will be skewed, non-parametric analysis through the Kruskal-Wallis test

Study outcomes will be compared between groups based on the intention-to-treat (ITT) principle. The change in CAIDE risk score from baseline vs. control will be analyzed using a mixed-effects model for repeated measures, adjusted for covariates as fixed effects. The covariates will be the baseline values of the covariates: age (years), education (years), sex (male vs. female), smoking status, and baseline CAIDE risk score. For each continuous endpoint, the baseline of the endpoint variable was included in the model. For genotype x intervention interaction effect, an interaction model will be created, and the influence will be evaluated through a generalized estimated equation model, wherein effect modification by the presence of ApoE 4 allele status will be analyzed by adding the group (yoga vs. walking) x time x variable interaction to the model, together with the main variable effect and variable x time and variable x group interactions. The model will also include interaction terms for treatment by month and a baseline CAIDE risk score by month. IBM SPSS 24.0 software will be used for all statistical analysis. Two-tailed tests will be used, and statistical significance will be set at a p-value < 0.05. Further, assuming an attrition rate of 20% and missing data, we also aim to conduct sensitivity analyses using the multiple imputation method. Subgroup analyses for age, gender, years of education, marital status, socio-economic class, BMI, SBP, Smoking and Alcohol status, etc.

The task of entering data will be carried out by clerical staff who have undergone training in research data entry. All participants will be assigned a participant number, and all data will be stored on an onsite server accessible only to the research team members. A range check will be performed for data values.

## Discussion

This paper presents the protocol for the DERREY Dementia Risk Reduction using yoga, a 48-week prospective longitudinal intervention study to evaluate the potential of a yoga-based program for dementia risk reduction. The available evidence on the risk-reducing potential of exercise or physical activity has been primarily derived from prospective cohort studies and case-control studies with a baseline measure of physical activity and a follow-up measure from all-cause dementia observational studies (40). Results from the FINGER trial with a multidomain intervention are supportive of the effect of the multidomain intervention on dementia risk reduction in older adults (41, 42). However, the findings were derived from post-hoc analysis. To our knowledge, the present trial would be one of the pioneer trials shedding insights into the potential of a lifestyle modification-based intervention for the prospective risk reduction of dementia. The study has also been designed with an adequate sample size to effectively capture the primary outcome, i.e., change in CAIDE risk scores, the main effect of the intervention. Since the study outcome CAIDE is an estimated risk score, and similar to the major recent trials, the FINGER study, the present trial targets at-risk individuals without substantial cognitive impairment, incident dementia has not been deemed as a feasible come after one year. However, targeting at-risk participants from the general population would aid in the direct translation of the findings for potential risk reduction in a public health context. If found effective, the study findings will aid in designing further longterm trials to actuate and extend the findings to the true reduction potential of yoga for the incidence of dementia.

The secondary outcome of the study will shed light on the aspect of genetic risk-based identification of yoga-based personalized intervention. To our knowledge, very few trial information can personalize lifestyle modification approaches to mitigate dementia risk. Some studies have reported that adhering to a healthy lifestyle may modify the risk reduction associated with ApoE ε4 allele status (41). However, we could find rare evidence of intervention-based studies that shed light on this aspect.

Another important attribute of the proposed trial is the inclusion of middle-aged individuals for dementia risk reduction, given the fact that the underlying modifiable vascular risk factors, such as national high blood pressure, smoking and obesity, are more predictive of cognitive decline at midlife compared to older age (43). Additionally, the study will also provide insight into the influence of yoga intervention on the quality of life and associated sleep problems in high-risk individuals that have been scarcely investigated in a longitudinal design. This study will have minimal side effects and a low cost of yoga intervention compared with other current treatments. The validated results will confirm the effectiveness of the protocol to be tested and tailored. One limitation of the trial is that it focuses on preventing dementia by using CAIDE risk scores to estimate risk reduction, which limits the direct clinical implications of the findings. However, recent reports support the association of CAIDE scores with the progression of both white mater hypertrophy and systemic inflammation in mid-life adults (44). Findings highlight the CAIDE score’s potential as both a prognostic and predictive marker in the context of cerebrovascular disease, identifying at-risk individuals who might benefit most from managing modifiable risk The CAIDE risk score can be used as a tool to communicate dementia risk and to select people who may benefit from lifestyle interventions. Based on the current results, it can perhaps also be used to track risk factor changes.

The major limitation of the study is the use of estimation-based risk reduction, using the CAIDE score, rather than the incidence of dementia. Another limitation is the limited follow-up time. However, findings from this study would serve as a proof-of-concept and pave a foundation for a larger study with long-term follow-up.

## Trial registration

CTRI/2023/02/049746.
